# Associations between participation in, intensity of, and time spent on leisure time physical activity and risk of inflammatory bowel disease among older adults (PA-IBD): a prospective cohort study

**DOI:** 10.1186/s12889-021-10492-7

**Published:** 2021-04-01

**Authors:** Nathalie Fogh Rasmussen, Bodil Hammer Bech, Katrine Hass Rubin, Vibeke Andersen

**Affiliations:** 1 Focused research unit for Molecular Diagnostic and Clinical Research (MOK), IRS-Center Sonderjylland, Hospital of Southern Jutland, Kresten Philipsens Vej 15 F, 6200 Aabenraa, Denmark; 2 Department of Public Health, Research Unit for Epidemiology, Aarhus University, Aarhus, Denmark; 3 OPEN - Open Patient data Explorative Network, Department of Clinical Research, University of Southern Denmark, and Odense University Hospital, Odense, Denmark; 4 Institute of Regional Health Research, University of Southern Denmark, Odense, Denmark

**Keywords:** Physical activity, Inflammatory bowel disease, Disease risk, Leisure time physical activity, Occupational physical activity, Cohort study

## Abstract

**Background:**

Inflammatory bowel diseases (IBDs) are diseases of the immune system that share some genetic and lifestyle-related predisposing factors. Increasing incidences have been reported in all age groups. Based on experimental studies suggesting a role of physical activity on intestinal inflammation, this study aimed to investigate the association between leisure time physical activity and the risk of IBD in older adults.

**Methods:**

The study is a prospective cohort study using Danish registry data and questionnaire data from the Danish “Diet, Cancer and Health” cohort. The outcome IBD was defined as having at least two main diagnoses of Crohn’s disease or ulcerative colitis registered in the National Patient Registry from the period between December 1993 and May 1997 with an average follow-up of 25 years. Cox proportional hazard models were used to estimate hazard-ratios for IBD onset associated with being physically active and with levels of the metabolic equivalent of task (MET) hours/week of physical activity and hours/week spent on six types of physical activity. All analyses were adjusted for potential confounders. Furthermore, the analyses were stratified according to age-group, occupational physical activity, smoking, BMI and work status to test for effect modification.

**Results:**

In total, 54,645 men and women aged between 50 and 64 years were included, and of which there were 529 cases. When comparing physically active with inactive participants measured by MET hours/week there was no statistically significant difference in risk of IBD (0.89 [0.13; 6.27]), regardless of how participation was measured. Results did not indicate any dose-response effect when comparing quartile groups of MET hours/week (HR = 0.97 [0.76; 1.22], HR = 0.82 [0.64; 1.05] and HR = 0.83 [0.65; 1.07] or whether five of the six types of activities were compared with the lowest quartile as reference. For do-it-yourself-work, the third quartile of hours/week was associated with a higher risk of IBD compared to the second quartile of hours/week (HR = 1.44 [1.10; 1.90]. No effect modification was found.

**Conclusions:**

There was no association between physical activity and risk of IBD when comparing physically active with inactive participants. Neither did the results indicate any dose-response effect when comparing quartile groups of MET hours/week with the lowest quartile as reference. Do-it-yourself work, however, appeared to be associated with a higher risk of IBD when comparing the third quartile with the second quartile of hours/week. The study has clinical relevance by its contribution to the explanatory field of the causes of IBD. However, the study has some limitations, and further research is needed to clarify associations between physical activity and risk of IBD.

**Supplementary Information:**

The online version contains supplementary material available at 10.1186/s12889-021-10492-7.

## Background

### Physical activity

Over the past two decades, there has been a consensus about the preventative and health promoting effects of physical activity (PA). The World Health Organization (WHO) states [1], PA contributes to various health benefits, such as reducing the risk of several noncommunicable diseases (NCDs) – diseases, which are a result of a combination of genetic, physiological, environmental and behavioural factors [2]. Chronic inflammatory bowel diseases (IBD) are an example of important NCDs that have emerged as a worldwide public health challenge, and therefore PA is highly relevant in a discussion of the prevention of these diseases.

### Chronic inflammatory bowel diseases

Crohn’s disease (CD) and ulcerative colitis (UC) are the two well-known forms of the IBDs. They are intestinal disorders resulting from an inappropriate inflammatory response to intestinal microbes [3, 4]. Production of pro-inflammatory cytokines by antigen-presenting cells may lead to a secretion of large amounts of tumour necrosis factor alpha (TNF-α). This further stimulates the activation of other pro-inflammatory responses, leading to a more permeable mucosal barrier which can result in microbial antigens from the intestinal lumen gaining access to the mucosal epithelium [5–8].

These diseases represent a public health problem because of their significant impact on patients and their families’ quality of life, on the health care system due to costly treatments, and on society due to absence from work. Furthermore, these diseases have challenging worldwide because of their high prevalence and incidence [9–11]. In general, research on IBD focuses on the occurrence of these diseases in early life (20–40 years of age), but the IBDs are also prevalent as late onset (40+ years of age) diseases [12–14]. Reported age-adjusted (45 to 69 years of age) incidences of the IBDs in Denmark are 6–10 for CD [13, 15] and 18–23 for UC [13, 15] per 100,000 person-years. Worldwide incidences are reported as up to 23 per 100,000 for CD and 57 per 100,000 for UC [10].

The detailed aetiology of the IBDs remains unknown, but experimental and observational studies such as meta-analyses and cohort- and case-control studies suggest that CD and UC have both genetic and environmental predisposing factors [16–20]. Use of nonsteroidal anti-inflammatory drugs (NSAIDs) [21], use of hormone replacement therapy (HRT) [22], obesity [23, 24] and dietary factors such as red meat [25] and alcohol [26, 27] have been suggested as risk factors for intestinal inflammation and onset of IBD, whilst it is suggested that intake of dietary fibre [16, 28] and fermented dairy products [29, 30] may play a preventative role. Interestingly smoking, has been associated with a higher risk of CD, whilst is seen as being protective for UC [31, 32].

### The preventative role of PA on the risk of IBD

Studies have also suggested a preventative effect of PA on the risk of IBD. Early studies have investigated the impact of occupational PA and found that occupations characterized by more physical work appeared to be protective compared with occupations characterized as sedentary [33, 34]. A study by Persson et al. investigating the impact of leisure time PA revealed that the relative risk (RR) of CD, was inversely related to weekly regular exercise (RR = 0.6 [0.4; 0.9]) [35].

Recent studies have reported contradictive results. The Nurses’ Health Study I and II [36] observed that women aged between 25 to 55 years who engaged in PA of more than 27 metabolic equivalents of task (MET) hours per week, had a 44% lower risk of developing CD compared with women who were inactive [36]. Chan et al. found that there was no association between PA and onset of IBD in “the European Prospective Investigation into Cancer and Nutrition” (EPIC) cohort which included people aged between 45 to 80 years old [37]. Furthermore, a meta-analysis including, among others, the aforementioned studies, reported that higher levels of PA were associated with a reduced risk of CD compared to those with low PA, but no association was found for UC [38].

**Fig. 1:**
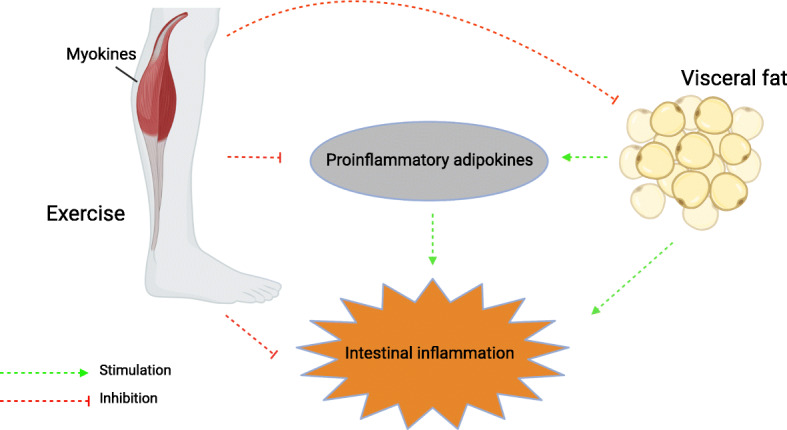
Crosstalk between skeletal muscle doing exercise, adipose tissue, and intestinal inflammation The hypothesis in this study is based on the suggested mechanism of crosstalk between skeletal muscle, adipose tissue and inflammation in the gut by Jan Bilski and colleagues [5]. Pathologically modified visceral adipose tissue has been demonstrated to secrete pro-inflammatory cytokines including TNF-a. Exercise may exert its anti-inflammatory response via a reduction in visceral fat mass and by inhibition of the secretion of pro-inflammatory cytokines, by releasing anti-inflammatory cytokines and myokines such as interleukin 6 (IL-6) from contracting muscles [5]. It has further been suggested that the effect of physical activity could depend on its intensity, duration, and type of exercise, with regular exercise being beneficial, while acute, strenuous exercise could lead to a release of inflammatory cytokines [5, 39]. But still, these mechanisms are not fully understood, and exercise such as running has also been found to induce increases in IL-6 [24, 40]. Note: Created with BioRender.com

The biological mechanisms supporting the hypothesis that being physically inactive compared to being active is associated with risk of IBD are based on a hypothesis described visually in Fig. 1 indicating `crosstalk´ between muscles, adipose tissue, and intestinal inflammation [5]. Furthermore, there is a growing body of evidence pointing at the role of PA in the modulation of gut microbiota towards a more diverse composition of the microbiome, which is associated with higher immunity [41]. The hypothesis is illustrated in Fig. 1.

Through these mechanisms of contracting muscles, adipose tissue, diversity of the gut microbiome, and intestinal inflammation, PA may play a protective role in the development of IBD.

### Aim

The aim of the present study was: *To investigate the association between PA and risk of IBD onset among older adults using leisure time PA as a proxy for PA*, by:

- operationalising leisure time PA as 1) intensity measured in MET hours, and 2) time spent on six different types of activities, to examine if a potential association may be primarily ascribed to specific activities.
- analysing intensity and time spent as A) being physically active compared to being inactive, and as B) a dose-response-association.

Furthermore, the study aimed to investigate whether the effect of leisure time PA was modified by the level of occupational PA, age, BMI and smoking habits.

## Methods

### Design and setting

A prospective cohort study design was used to investigate the described hypothesis. The study was based on data from the Danish “Diet, Cancer and Health” (DCH) cohort and from Danish registries. Participants in the DCH cohort were recruited between December 1993 and May 1997, and included a total of 57,053 participants (27,178 men and 29,875 women) aged between 50 and 64 years of age, with residence in the areas of either Copenhagen or Aarhus, born in Denmark and with no previous cancer diagnosis in the Danish Cancer Registry. At baseline, the participants completed a lifestyle questionnaire and a food frequency questionnaire (FFQ). Both questionnaires were interviewer-checked and validated regarding PA and diet [42, 43]. A detailed description of the DCH cohort has been described elsewhere [44]. This questionnaire developed for the DCH cohort is available from the Danish Cancer Society [45].

### Participants, eligibility criteria and follow-up

#### Inclusion criteria

All men and women included in the DCH cohort with no diagnosis of CD or UC (diagnostic codes described in next section) before entry to the cohort and with information on PA were included in the analyses.

*Follow-up:* The participants were followed from the date of their first visit at the DCH study clinic until either date of diagnosis of CD or UC, date of death or emigration, or the date December 31, 2018, whichever came first.

### Materials, data sources, and methods

#### Outcome diagnostic criteria

The outcome late onset IBD was defined by the criteria: 1) having a main diagnosis (A-diagnosis) registered in the Danish National Patient Registry (DNPR) [46] with the International Classification of Diseases (ICD) 8 and 10 codes for CD (563.00–563.09, 563.91 and K50 (including all sub-codes)) and UC (563.19, 563.99, 569.04, and K51 (including all sub-codes)) in years 1977–2018 from a department with a relevant area of specialization (Surgical Gastroenterology, Medical Gastroenterology, Internal medicine) and 2) the diagnosis was followed by at least one additional registration in the DNPR (inpatient or outpatient visit) related to the first diagnosis within 180 days. The date and year of the diagnosis were defined as the date and year of the first diagnosis registered in the DNPR.

#### Registry data

The Danish health registries included the DNPR and the Danish Civil Registration System (DCRS) [47]. The DNPR was used to identify patients with IBD during follow-up. ICD-8 and ICD-10 codes from the DNPR were used to identify cases diagnosed before and after entry to the DCH cohort, and to calculate comorbidity in the cohort using the updated Charlson’s comorbidity index [48]. Comorbidity was categorized as a binary variable (comorbidity = no/yes) to ensure enough power of the group with comorbidity. High completeness of IBD registration in the DNPR has previously been reported(94%), with an estimated positive predictive value of 97% for CD and 90% for UC [49]. The DCRS was used to extract follow-up information on death and immigration. Data were linked by the unique identification number assigned to all residents in Denmark at birth or first immigration.

#### Exposure

The exposure was defined as 1) a binary indicator of exposure: being active/inactive, both for total intensity of PA and separated on the six different types of leisure time PA: walking, housework, gardening, do-it-yourself work, cycling and sports, and as 2) levels of intensity of total PA and time spend on the six types of activities. The intensity was measured as MET hours/week. The time spent was measured as hours/week.

The MET system is based on the understanding that all activities are assigned an intensity unit based on their rate of energy expenditure. One MET is defined as the energy expenditure at rest (the resting metabolic rate), which for the average adult is approximately 3.5 ml of O_2_/kg body weight/min. The intensities of different activities are calculated as the ratio between the associated metabolic rate for the specific activity and the resting metabolic rate [50].

Being active was defined as having an intensity level of ≥3 MET hours/week or as spending > 0 h/week on each type of activity. The cut point for the binary variable of MET hours/week was chosen to correspond to inactivity equivalent of < 1 h of walking at an average pace per week, consistent with prior studies [36]. The levels of intensity and time spent were categorised in quartiles. A pre-defined variable for MET hours/week from the DCH dataset was used. The variable is further described below.

#### Questionnaire data

Information on leisure time PA was based on six questions covering the average number of hours per week spent over the past year on the six types of leisure time PA during summer and winter, respectively. The MET hours/week variable was calculated by multiplying the MET value of each specific activity by duration and frequency of the activities. The following MET-values were used according to Ainsworth’s *Compendium of Physical Activities* [50, 51]: walking 3.0, housework 3.0, gardening 4.0, do-it-yourself work 4.5, cycling 6.0, and sports 6.0.

#### Covariates and potential confounders

Based on the known and commonly accepted IBD risk and preventative factors, the analyses were adjusted for occupational PA, smoking, intake of fibre, fermented dairy products, red and processed meat, alcohol, HRT (only for women), NSAID, comorbidity, and also the demographic factors age and gender. These factors were expected to be possible confounders of the association between leisure time PA and risk of IBD.

Information on occupational PA was obtained from a question with five categories (sitting, standing, light manual work, heavy manual work, no occupation). Light and heavy manual work were combined in one category. Total energy intake was measured in mega joule (MJ) per day, alcohol consumption and intake of fibre, fermented dairy products and meat (red and processed meat) were measured in grams per day – all retrieved from the FFQ. A detailed description of the calculation of the dietary variables in the DCH study is described elsewhere [52]. Smoking habits within the past year were defined as current, never or former. The questionnaire also gave information on the use of a pain-relieving medicine, which was defined by the variable NSAID and assessed as > 1 tablet per month during the last year before baseline (yes/no). HRT was divided into the following categories: never, current, and former user.

### Statistical analyses

To investigate the risk of and time to an IBD event the Cox proportional hazards model with age as the underlying time scale was applied. Death, emigration and loss to follow-up were not considered as competing risks, and thus, were managed with censoring. The assumption of proportional hazards was checked by evaluating parallel curves of the cumulative hazard function on the log-scale. Furthermore, sensitivity analyses modelling time-varying effects of covariates that did not fulfil the proportional hazard assumption, were performed.

Hazard-ratios (HRs) and the corresponding 95% confidence intervals (95% CI) and *p*-values for IBD onset associated with participation in and levels of leisure time PA were estimated. Analyses were also separated on CD and UC, and due to the limited number of cases in each group, these analyses only included age, gender, occupational physical activity, smoking, and comorbidity as adjusting factors based on the literature of known risk factors. All analyses were carried out according to the principle of complete-case-analysis [53] to ensure an equal number of participants in all analyses. The analyses were performed for both a crude model and a model adjusted for baseline values of preventative and risk factors of IBD. In the dose-response analyses, inactive individuals were included by assigning indicator variables of being active/inactive, as the IBD risk among inactive individuals may deviate from the risk among active individuals. Since the lowest quartile group of gardening, do-it-yourself-work, cycling, and sports only included inactive people (0 h/week), the second quartile group was used as a reference for these variables.

Furthermore, the analyses were stratified according to strata of age groups (50–59 and 60–64 years), BMI (< 25 kg/m^2^ and ≥ 25 kg/m^2)^, smoking (‘never smoker’ and ‘current/former smoker’), occupational PA (‘not active at work’, including sitting and not working, and ‘active at work’, including standing and manual work) and work status (not working/working), as these variables were assumed to interact with the effect of leisure time PA.

All analyses were carried out using *Stata version 15* [54]. For all tests, a *p*-value below 0.05 was considered statistically significant.

The study was not submitted to the Southern Denmark Ethics Committee. The study does not need approval from an Ethics committee or Institutional Review Board as according to Danish law: “*Questionnaire studies and health science registry research projects must be reported to the scientific ethics committee system only if the project includes human biological material*” (the Act on Research Ethics Review of Health Research Projects) [55].

## Results

**Fig. 2:**
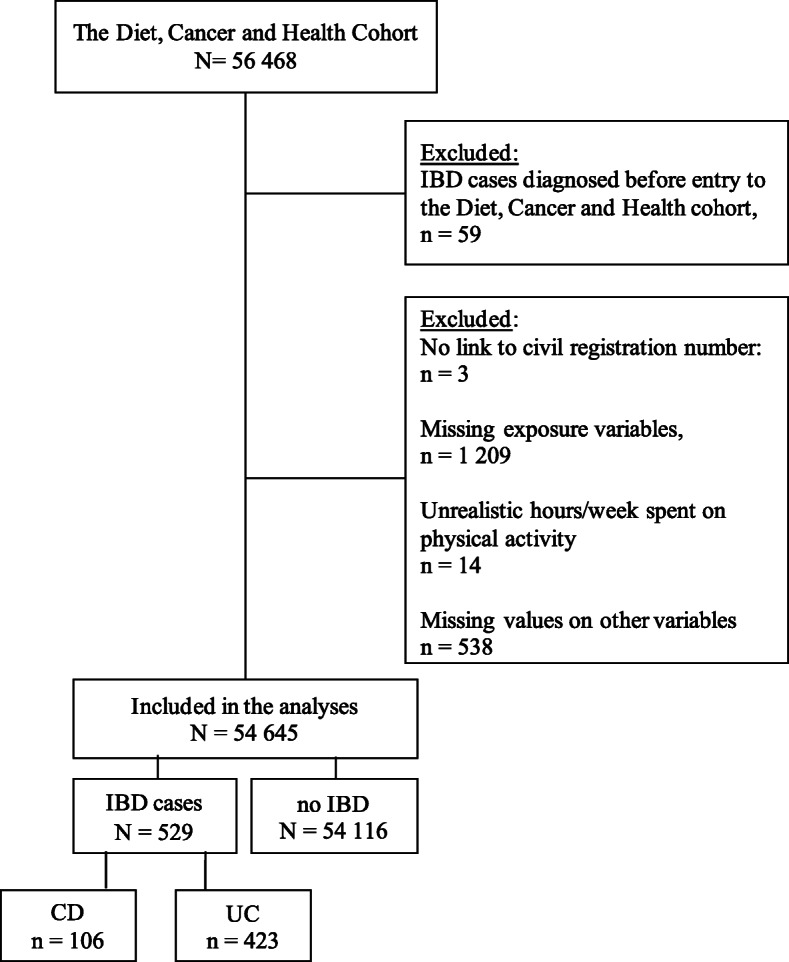
Flowchart of the study population. Note: Some patients (*N* = 23) had received a diagnosis of both CD and UC during their disease course. These patients were classified according to the last diagnosis as the last diagnosis registered was regarded as the most valid, knowing that some patients change diagnosis during their disease follow-up

In total, 1823 individuals were excluded from the study cohort; 59 participants were excluded because they had a CD or UC diagnosis before entry to the DCH cohort; three individuals where excluded because of no link to the DCRS; 1209 were excluded because of missing values in one or more of the PA variables, and 14 were excluded due to implausibly lengthy timespan recorded on leisure time PA (> 105 h/week), which was in accordance with previous studies using the DCH cohort [56, 57]. The remaining 538 individuals were excluded because of missing information on other variables. A total of 28,526 women and 26,119 men were included in the analyses. During a mean follow-up of 25 years, 529 IBD cases (106 CD cases and 423 UC) were observed (Fig. 2).

The median MET hours in each quartile group were as follows: 1st quartile: 27 h/week; 2nd quartile: 47 h/week; 3rd quartile: 69 h/week, and 4th quartile: 112 h/week (Table 1). Table 1 also presents the medians and 25th and 75th percentiles of hours spent on the six types of activities in each quartile group.

**Table 1:** Median and percentiles of MET hours and hours per week in quartile groups

Dose-response of physical activity	Quartiles of physical activity (hours/week)			
	1st median (p1; p3)	2nd median (p1; p3)	3rd median (p1; p3)	4th median (p1; p3)
MET hours/week	27 (20; 32)	47 (42; 52)	69 (63; 76)	112 (96; 140)
Total activity	7 (6; 9)	12 (11; 14)	18 (16; 20)	29 (25; 36)
Walking	1 (0.5; 1.5)	2.5 (2; 3)	4 (4; 5)	8 (7; 10.5)
Housework	1 (1; 2)	3 (3; 4)	6 (5; 6)	10 (10; 15)
Gardening	0	1 (0.5; 1.5)	2.5 (2; 3)	5 (4; 7.5)
Do-it-yourself-work	0	1 (1; 1)	2 (1.5: 2)	4.5 (3; 7.5)
Cycling	0	1 (0.5; 1)	2 (1.5; 2.5)	5.5 (4; 7.5)
Sports	0	0.5 (0.5; 0.5)	1 (1; 2)	4 (3; 5)

*Abbreviations*: *MET* metabolic equivalent of task, *p1; p3* 25th and 75th percentiles

Baseline characteristics of the cohort grouped in quartiles of MET hours of PA per week are presented in Table 2. Due to asymmetric distributions, continuous variables were presented as medians with interquartile ranges. The groups differ on several parameters. A higher proportion of the youngest age group (50–54 years) was represented in each MET quartile group, but the proportion was higher in the lowest quartile group compared to the highest quartile group. A higher proportion of women, were represented in all MET quartile groups except the lowest quartile group. A markedly higher proportion of people not working and a lower proportion of people with sedentary work were represented in the highest MET quartile group compared to the distribution in the lowest MET group.

**Table 2:** Baseline characteristics of participants in quartile groups of MET hours/week

*N* study population = 54,645	Quartile groups of MET hours/week			
	1st (*N* = 13,827)	2nd (*N* = 13,554)	3rd (*N* = 13,720)	4th (*N* = 13,544)
MET hours/week^a^	27 (20; 32)	47 (42; 52)	69 (63; 76)	112 (96; 140)
Age	56 (53; 60)	56 (53; 60)	56 (53; 60)	57 (53; 61)
Age groups				
50–54 years	6084 (44)	6057 (45)	5907 (43)	5116 (38)
55–59 years	4330 (31)	4214 (31)	4221 (31)	4156 (31)
60–64 years	3413 (25)	3283 (24)	3592 (26)	4272 (31)
Gender				
Women	6476 (47)	7125 (53)	7548 (55)	7377 (54)
Men	7351 (53)	6429 (47)	6172 (45)	6167 (46)
Physical activity at work				
Sedentary	5894 (43)	5598 (41)	4910 (36)	3254 (24)
Standing	2382 (17)	2425 (18)	2348 (17)	2254 (17)
Manual	3089 (22)	3142 (23)	3413 (25)	3895 (29)
Not working	2462 (18)	2389 (18)	3049 (22)	4141 (31)
Dietary factors				
Energy (MJ/d),	9.2 (7.5; 11.0)	9.4 (7.7; 11.0)	9.6 (8.0; 11.0)	10.0 (8.3; 12.0)
Dietary fibre intake (g/d)	19 (15; 23)	20 (16; 25)	21 (17; 26)	22 (17; 27)
Meat intake (g/d)	109 (78; 148)	105 (76; 143)	105 (75; 143)	107 (77; 150)
Fermented dairy products	38 (12; 176)	53 (16; 198)	60 (18; 204)	68 (19; 205)
Alcohol intake (g/d)	13 (6; 31)	13 (6; 31)	13 (6; 31)	13 (5; 31)
Smoking				
Never	4588 (33)	4881 (36)	5068 (37)	4732 (35)
Former	3840 (28)	4011 (30)	4075 (30)	3864 (28)
Current	5399 (39)	4662 (34)	4577 (33)	4948 (37)
BMI (kg/m^2^)				
< 25.0	5407 (39)	6015 (44)	6201 (45)	6025 (44)
≥ 25	8420 (61)	7539 (56)	7519 (55)	7519 (56)
Comorbidity				
No (CCI = 0)	13,300 (96)	13,151 (97)	13,306 (97)	13,078 (97)
Yes (CCI = ≥ 1)	527 (4)	403 (3)	414 (3)	466 (3)
NSAID				
No	9337 (68)	9075 (67)	9248 (67)	9155 (68)
Yes (> 1 pill/month)	4490 (32)	4479 (33)	4472 (33)	4389 (32)
HRT (women, *N* = 28,406)				
Never	3547 (55)	3914 (54)	4065 (54)	4013 (54)
Current	1954 (30)	2113 (30)	2287 (30)	2193 (30)
Former	975 (15)	1098 (15)	1196 (16)	1171 (16)

^a^ Median and 25th and 75th percentiles (p1; p3) are presented for continuous variables. Number, N, and percent (%) are presented for categorical variables. All values after exclusion of missings. Abbreviations: *MET* metabolic equivalent of task, *g/d* gram per day, *BMI* body mass index, *CCI* Charlson’s comorbidity index, *HRT* hormone replacement therapy, *NSAID* nonsteroidal anti-inflammatory drugs

### Active/inactive

An intensity of ≥3 MET hours/week (indicator of being active) was not associated with any significantly lower risk of IBD (0.89 [0.13; 6.27]) after adjustment for potential confounders. In addition, engaging in any of the six types of leisure time activities was similarly not associated with a lower risk of IBD (Table 3).

**Table 3:** Risk of inflammatory bowel disease according to being physically active

Indicator variables of physical activity	Crude		Adjusted^a^	
	HR^b^	95% CI	HR^b^	95% CI
MET hours/week				
Active (> 3 MET hours/week)	0.77	(0.11; 5.49)	0.89	(0.13; 6.27)
Active (> 0 h/week) in each activity				
Walking	1.02	(0.73; 1.44)	1.07	(0.76; 1.52)
Housework	1.03	(0.72; 1.47)	1.04	(0.72; 1.50)
Gardening	0.87	(0.72; 1.05)	0.97	(0.79; 1.19)
Do-it-yourself work	0.87	(0.73; 1.03)	0.92	(0.75; 1.14)
Cycling	0.87	(0.73; 1.04)	0.93	(0.77; 1.12)
Sports	0.92	(0.78; 1.09)	1.02	(0.85; 1.22)

^a^Adjusted for age, gender, occupational physical activity, smoking, energy intake, intake of meat, fibre, fermented dairy products and alcohol, nonsteroidal anti-inflammatory drugs, hormone replacement therapy, comorbidity. The six types of activity were mutually adjusted

^b^Inactive is the reference for all estimates

*Abbreviations*: *MET* metabolic equivalent of task, *HR* hazard ratio, *CI* confidence interval

### Dose-response

Compared with participants in the lowest quartile of MET hours/week, there were no statistically significantly higher or lower risk of IBD with increasing MET hours/week, neither when separating the analyses on CD and UC (Tables 4 and 5). Furthermore, no statistically significant associations were found for quartiles of walking, housework, gardening, cycling and sports. For do-it-yourself-work, the unadjusted HRs indicated that the lowest and the third quartiles were associated with a higher risk of IBD compared to the second quartile of hours/week (HR = 1.27 [1.01; 1.59] and HR = 1.43 [1.09; 1.86]). Furthermore, in the adjusted analyses the association remained significant for the third quartile (HR = 1.44 [1.10; 1.90] (Table 4).

**Table 4:** Risk of inflammatory bowel disease according to quartiles of physical activity

Quartile groups of physical activity	Crude		Adjusted^a^	
	HR^b^	95% CI	HR^b^	95% CI
MET hours/week				
2nd quartile	0.94	(0.75; 1.19)	0.97	(0.76; 1.22)
3rd quartile	0.81	(0.63; 1.03)	0.82	(0.64; 1.05)
4th quartile	0.86	(0.68: 1.09)	0.83	(0.65; 1.07)
Walking (hours/week)				
2nd quartile	0.89	(0.71; 1.11)	0.86	(0.68; 1.09)
3rd quartile	0.87	(0.68; 1.13)	0.83	(0.63; 1.08)
4th quartile	0.96	(0.76; 1.21)	0.86	(0.67; 1.11)
Housework (hours/week)				
2nd quartile	1.10	(0.88; 1.37)	1.12	(0.88; 1.43)
3rd quartile	0.95	(0.74; 1.22)	0.97	(0.74; 1.29)
4th quartile	1.14	(0.91; 1.44)	1.12	(0.84; 1.47)
Gardening (hours/week)				
1st quartile	1.10	(0.88; 1.36)	0.98	(0.78; 1.24)
3rd quartile	0.82	(0.64; 1.06)	0.81	(0.62; 1.04)
4th quartile	1.01	(0.80; 1.28)	0.95	(0.74; 1.23)
Do-it-yourself (hours/week)				
1st quartile	1.27	(1.01; 1.59)	1.17	(0.92; 1.49)
3rd quartile	1.43	(1.09; 1.86)	1.44	(1.10; 1.90)
4th quartile	0.99	(0.76; 1.29)	0.97	(0.73; 1.29)
Cycling (hours/week)				
1st quartile	1.04	(0.83; 1.31)	0.97	(0.77; 1.23)
3rd quartile	0.91	(0.70; 1.16)	0.91	(0.70; 1.17)
4th quartile	0.82	(0.63; 1.07)	0.83	(0.64; 1.09)
Sports (hours/week)				
1st quartile	1.03	(0.72; 1.50)	0.94	(0.66; 1.36)
3rd quartile	0.94	(0.64; 1.37)	0.92	(0.63; 1.35)
4th quartile	0.96	(0.64; 1.43)	0.99	(0.66; 1.48)

^a^Adjusted for age, gender, occupational physical activity, smoking, energy intake, intake of meat, fibre, fermented dairy products and alcohol, nonsteroidal anti-inflammatory drugs, hormone replacement therapy, comorbidity. The six types of activity were mutually adjusted

^b^1st quartile is the reference for MET hours, walking and housework. 2nd quartile is reference for gardening, do-it-yourself, cycling and sports

*Abbreviations*: *MET* metabolic equivalent of task, *HR* hazard ratio, *CI* confidence interval

**Table 5:** Risk of Crohn’s disease and ulcerative colitis according to quartiles of physical activity

Quartile groups of physical activity	Crude		Adjusted^a^	
	HR^b^	95% CI	HR^b^	95% CI
*Crohn’s disease*				
MET hours/week				
2nd quartile	0.65	(0.38; 1.13)	0.67	(0.39; 1.17)
3rd quartile	0.93	(0.56; 1.52)	0.93	(0.56; 1.54)
4th quartile	0.74	(0.43: 1.26)	0.69	(0.40; 1.20)
*Ulcerative colitis*				
MET hours/week				
2nd quartile	1.02	(0.79; 1.32)	1.02	(0.80; 1.34)
3rd quartile	0.77	(0.59; 1.02)	0.78	(0.59; 1.02)
4th quartile	0.89	(0.68: 1.16)	0.86	(0.66: 1.13)

^a^Adjusted for age, gender, occupational physical activity, smoking, comorbidity

^b^1st quartile is the reference

*Abbreviations*: *MET* metabolic equivalent of task, *HR* hazard ratio, *CI* confidence interval

### Stratification

The results of the stratified analyses indicated that the dose-response estimates were not modified by age (50-59 and 60-64), occupational PA (sedentary/not working and standing/manual work), BMI (< 25.0 and ≥ 25.0), smoking (never and former/current) or work status (not working and working). All P for *z*-tests> 0.05. (Supplemental Table S1).

## Discussion

This cohort study of Danish middle-aged men and women did not find any support for the hypothesis that being physically active compared to being inactive measured by MET hours/week and as participation in six types of activities lowered the risk of IBD. Generally, estimates had wide confidence intervals. In separate analyses for UC and CD, the study neither found any statistically significant associations.

### Comparison with other studies

The findings of this study may to some extent be compared with those of the EPIC cohort, which did not observe an association between PA and risk of CD and UC [37]. Compared with the present study, the EPIC study (which included participants from the DCH cohort) only included 75 CD cases and 177 UC cases and was also unable to account for long-term changes in PA.

A combined study of the Nurses’ Health studies I and II used detailed and updated information on PA and known risk factors for CD and UC and included 284 CD cases and 363 UC cases. They reported a HR of 0.56 (0.37; 0.84) for the risk of developing CD when comparing active women with at least 27 MET hours/week of PA with sedentary women with < 3 MET hours/week. No association was found for UC. Age, smoking, and BMI did not significantly modify the association between PA and risk of UC or CD. Furthermore, the findings from the Nurse’s Health studies are consistent with findings from two prior case-control studies [35, 58]. Persson et al. used a mailed questionnaire to obtain information on PA from cases and controls and reported an inverse association between weekly regular PA and risk of CD but not UC [35]. Klein et al. found that IBD patients had lower levels of PA during their pre-illness period than clinical controls [58].

A meta-analysis by Wang et al. [38] argues that the variations in definitions of PA across studies make comparisons difficult. The Nurse’s health study used MET hours/week and therefore was more comparable with our study compared to the EPIC study, where a PA index combining occupational and recreational PA was used. However, the median of MET hours/week in the highest quartile group in this study was 112 h/week compared to only 45 h/week in the Nurse’s Health study. Thus, this study population generally reported very high numbers of hours spent on leisure time PA, complicating the comparability. The Nurse’s Health study found an effect at 27 MET hours/week when compared to the lowest category of PA (< 3 MET hours/week). If the effect of PA has a threshold value the effect is probably not shown in this study, as this study uses a higher PA level as reference group (27 MET hours/week) and not the < 3 MET hours/week as in the Nurse’s Health study. We accepted a maximum of 105 h/week. However, a maximum of 60 h/week may be more realistic, taking 37 h work per week into account. A supplemental analysis, where individuals with > 60 h PA/week were excluded (368 individuals), and a grouping of MET level in fifths instead of fourths was used, lead to similar results. This can be explained by the reported PA levels that remained much higher than the reported levels in the NHS, and thus, the low number of excluded individuals.

Wang et al. found an association between high PA and decreased risk of developing CD. In contrast, there was no significant inverse association between PA and UC. Other studies have suggested that PA in the pre-illness period is associated with reduced risk of the onset of IBD, revealing this association being much stronger for CD than UC [57]. Thus, these studies could indicate a difference in response to PA in the two diseases, which is also seen in the sub-analyses of dose-response in this study (Table 5), even though results were not statistically significant. However, the analysis for CD was based on very few cases in each quartile group, resulting in broad confidence intervals.

### Confounders and factors related to physical activity

Several factors were expected to be possible confounders of the association between leisure time PA and risk of IBD, as these factors are all related to IBD as mentioned in the background section, and expected to be related to PA. PA level decreases with age, and the type of PA may differ between men and women [59, 60]. Dietary habits, alcohol intake as well as smoking habits may be associated with a particular lifestyle, health behaviour and socioeconomic status, and thus specific PA habits [61]. NSAIDs may be taken due to pain which may affect the level of PA. Sex hormones may have an influence on activity levels [62], and comorbidities such as cardiovascular diseases may also be associated with being physically active or inactive, due to either disease-related recommendations of PA or impaired physical functioning [63].

Furthermore, sleep disorder and IBD appear to be related in a bidirectional way [64]. It is therefore of interest to know whether there is any interaction between sleep disorder and PA. Unfortunately, data on sleep habit were not available in the DCH questionnaire. Thus, this relevant interaction was not possible to analyse in this study.

### Strengths and limitations

The present study has several strengths. A major strength is its prospective design, which reduced selection bias. As the participants were followed using registries, the loss to follow-up was minimal. Information on PA and covariates were measured several years before the onset of disease, and hence, were unrelated to IBD status later during follow-up. Another major strength is the linkage to the DNPR, which is considered of high validity and completeness, and the restrictive diagnostic criteria by including only A-diagnoses and cases with at least two diagnoses during follow-up. This approach ensured that a high proportion of the identified cases really had IBD, hence increasing the specificity but lowering the sensitivity, as some ‘real’ IBD cases might not have been identified. Moreover, the diagnostic criteria were in accordance with the criteria used in a Danish nationwide cohort study of IBD using data from the DNPR [13].

The study had three notable limitations. *Firstly*, the available data did not allow exploration of the association between timing in life of PA and risk of IBD. It could be argued that a potential protective effect of PA is due to the accumulated PA exposure during life. Whether the PA level reported at baseline is representative of the PA level throughout life can be questioned. The included age group may be characterised by substantial lifestyle changes including retirement and development of comorbidities other than those at baseline. These lifestyle changes may affect PA habits. However, the median time to IBD diagnosis was 8 years, which may not include major lifestyle changes as most people are not yet retired. None the less, the inability of this study to account for long-term changes of PA must be perceived as a major limitation.

*Secondly*, there were only 106 CD and 423 UC cases. These numbers are higher than in the EPIC study, but still limit the opportunity to look at the diseases separately, although the study did separate the dose-response analyses on CD and UC. The low number of cases can be explained by the restriction to the age group 50–64 years, which is not the age of typical IBD onset (20–30 years) [13]. However, 50–64 years is an age group with increasing IBD incidence [12–14].

*Thirdly*, although the DCH questionnaire is demonstrated to be a reliable and valid tool [43], the limitations according to self-reported information should be considered. PA is, in general, difficult to measure accurately in observational studies as there is a risk of measurement error and misclassification. The participants had generally reported very high levels of leisure time PA per week equivalent of up to 15 h/day, hence it is possible that PA may have been overreported. This must have been independent of the outcome and may have resulted in bias towards the null hypothesis in the analysis of the dichotomised exposure variables. In the analyses with the exposure in quartiles, the misclassification resulted in a risk of both over- and underestimation of the association. Overall, the results of the present study should be interpreted with caution.

Although the present study cannot explicitly conclude that individuals are likely reduce their risk of IBD through participation in PA, there are plausible biological mechanisms for how PA may be involved in the aetiology of IBD. These mechanisms originate until now from evidence from experimental animal and human studies suggesting that PA may exert an anti-inflammatory effect via a reduction in visceral fat mass and/or by induction of an anti-inflammatory intestinal environment [5, 6]. Therefore, there is a rationale for future cohort studies to further investigate this association. Further research is needed to clarify the association which is not fully understood by the present study.

## Conclusion

In conclusion, this study did not find any association between intensity of and time spent on PA and risk of IBD when comparing physically active and inactive participants. Neither did the results indicate any dose-response effect when comparing quartile groups of MET hours/week (neither IBD nor the separated analyses on CD and UC) or hours/week spent on specific activities (analyses on IBD). However, do-it-yourself work, appeared to be associated with a higher risk of IBD when comparing the third quartile with the second quartile of hours/week. The five other activities (walking, housework, cycling, gardening and sport) did not show any significant associations. The study has important public health implications in a society dominated by a sedentary lifestyle, and clinical relevance by its contribution to the explanatory field of the causes of IBD.

## Supplementary Information

**Additional file 1: Supplemental Table S1.** Hazard ratios from adjusted analyses stratified on age, smoking, BMI, physical activity at work, and work status. Results of the analyses stratified according to strata of age groups (50–59 and 60–64 years), BMI (< 25 kg/m^2^ and ≥ 25 kg/m^2)^, smoking (‘never smoker’ and ‘current/former smoker’), occupational PA (‘not active at work’, including sitting and not working, and ‘active at work’, including standing and manual work) and work status (not working/working).

## Data Availability

The data that support the findings of this study are available from Open Patient data Explorative Network (OPEN) but restrictions apply to the availability of these data, which were used under license for the current study, and so are not publicly available. Data are however available from the authors upon reasonable request and with permission of the Danish Health Data Authority. Permissions obtained to access data before the initiation of this project were obtained from The Danish Data Protection Agency, and furthermore from the data owners: The Danish Cancer Society and the Danish Health Data Authority.

## Abbreviations

PA: Physical activity
IBD: Inflammatory bowel disease
CD: Crohn’s disease
UC: Ulcerative colitis
DCH: Diet, Cancer and Health
EPIC: European Prospective Investigation into Cancer and Nutrition
MET: Metabolic equivalent of task
NSAID: Nonsteroidal anti-inflammatory drugs
HRT: Hormone replacement therapy
HR: Hazard ratio
95% CI: 95% confidence interval
p1 and p3: 25th and 75th percentiles
