# Supplementary material for: Associations between participation in, intensity of, and time spent on leisure time physical activity and risk of inflammatory bowel disease among older adults (PA-IBD): a prospective cohort study
Source: BMC Public Health. 2021 Apr 1;21:634. doi: 10.1186/s12889-021-10492-7 (PMC8015056; doi:10.1186/s12889-021-10492-7)
Supplement: Supplementary file 1 — Additional file 1: Supplemental Table S1. Hazard ratios from adjusted analyses stratified on age, smoking, BMI, physical activity at work, and work status. Results of the analyses stratified according to strata of age groups (50–59 and 60–64 years), BMI (< 25 kg/m2 and ≥ 25 kg/m2), smoking (‘never smoker’ and ‘current/former smoker’), occupational PA (‘not active at work’, including sitting and not working, and ‘active at work’, including standing and manual work) and work status (not working/working). [file 12889_2021_10492_MOESM1_ESM.pdf]

Supplemental table S1. Hazard ratios from adjusted analyses stratified on age, smoking, BMI, physical activity at work, and work status

	MET hours			Walking			Housework			Gardening			Do-it-yourself			Cycling			Sport		
2nd quartile																					
50-59 years	**0.99**	0.70	1.41	**0.72**	0.50	1.03	**0.94**	0.64	1.37	**1.07**	0.77	1.48	**0.93**	0.64	1.34	**1.16**	0.80	1.68	**1.13**	0.60	2.12
60-64 years	**0.71**	0.44	1.15	**0.94**	0.37	1.16	**1.13**	0.68	1.90	**0.44**	0.24	0.81	**1.56**	0.93	2.61	**0.92**	0.57	1.46	**1.00**	0.48	2.10
Non smoker	**1.24**	0.70	2.21	**1.15**	0.65	2.04	**0.99**	0.51	1.91	**1.10**	0.63	1.90	**1.32**	0.74	2.36	**0.71**	0.39	1.28	**1.30**	0.51	3.34
Smoker	**0.79**	0.57	1.10	**0.71**	0.51	0.99	**1.01**	0.72	1.43	**0.80**	0.58	1.12	**1.06**	0.74	1.51	**1.21**	0.86	1.70	**1.02**	0.58	1.78
BMI <25.0	**1.10**	0.71	1.72	**0.66**	0.42	1.05	**1.15**	0.72	1.83	**1.17**	0.76	1.81	**1.14**	0.72	1.78	**0.79**	0.50	1.24	**0.57**	0.31	1.03
BMI >25.0	**0.77**	0.53	1.12	**0.90**	0.63	1.30	**0.90**	0.60	1.36	**0.66**	0.45	0.97	**1.10**	0.73	1.65	**1.30**	0.88	1.92	**2.38**	0.97	5.86
Sedentary	**0.79**	0.55	1.12	**0.89**	0.62	1.28	**1.19**	0.82	1.75	**0.83**	0.58	1.19	**1.02**	0.71	1.46	**1.18**	0.83	1.67	**1.06**	0.58	1.94
Active at work	**1.11**	0.69	1.80	**0.69**	0.43	1.10	**0.73**	0.43	1.23	**0.94**	0.59	1.49	**1.42**	0.84	2.39	**0.82**	0.48	1.39	**1.09**	0.49	2.39
Not working	**0.85**	0.44	1.63	**0.85**	0.43	1.67	**1.77**	0.87	3.62	**0.52**	0.29	0.93	**1.16**	0.64	2.10	**0.87**	0.47	1.60	**0.72**	0.28	1.87
Working	**0.89**	0.65	1.22	**0.79**	0.58	1.08	**0.85**	0.60	1.20	**1.02**	0.74	1.41	**1.10**	0.78	1.56	**1.13**	0.81	1.58	**1.18**	0.68	2.07
3rd quartile																					
50-59 years	**0.68**	0.46	1.02	**0.78**	0.52	1.18	**0.77**	0.49	1.21	**0.76**	0.52	1.12	**1.10**	0.72	1.68	**1.06**	0.71	1.59	**1.14**	0.60	2.16
60-64 years	**0.74**	0.47	1.18	**0.66**	0.37	1.16	**1.14**	0.87	2.60	**0.44**	0.23	0.84	**1.92**	1.11	3.31	**1.00**	0.61	1.65	**0.87**	0.40	1.88
Non smoker	**1.13**	0.63	2.04	**1.20**	0.63	2.27	**1.23**	0.61	2.45	**0.62**	0.31	1.25	**1.56**	0.79	3.07	**0.94**	0.52	1.68	**1.09**	0.42	2.84
Smoker	**0.60**	0.42	0.86	**0.62**	0.42	0.92	**0.79**	0.52	1.21	**0.68**	0.47	0.99	**1.30**	0.89	1.90	**1.09**	0.76	1.59	**1.01**	0.57	1.80
BMI <25.0	**0.88**	0.55	1.42	**0.95**	0.59	1.53	**1.03**	0.61	1.74	**0.71**	0.42	1.20	**1.23**	0.72	2.11	**0.90**	0.57	1.43	**0.67**	0.37	1.22
BMI >25.0	**0.63**	0.42	0.94	**0.58**	0.36	0.93	**0.79**	0.49	1.29	**0.64**	0.42	0.97	**1.42**	0.93	2.17	**1.18**	0.77	1.81	**2.01**	0.80	5.03
Sedentary	**0.60**	0.41	0.89	**0.80**	0.53	1.22	**0.98**	0.63	1.54	**0.71**	0.47	1.08	**1.16**	0.76	1.77	**0.83**	0.55	1.25	**1.09**	0.59	2.02
Active at work	**0.98**	0.60	1.59	**0.67**	0.39	1.16	**0.76**	0.42	1.39	**0.62**	0.36	1.06	**1.72**	1.00	2.95	**1.47**	0.90	2.42	**0.91**	0.40	2.07
Not working	**0.63**	0.32	1.22	**0.91**	0.44	1.88	**1.39**	0.64	3.05	**0.51**	0.25	1.03	**1.15**	0.54	2.44	**0.69**	0.36	1.34	**0.76**	0.29	2.01
Working	**0.74**	0.53	1.04	**0.70**	0.48	1.02	**0.78**	0.52	1.18	**0.71**	0.49	1.03	**1.42**	0.98	2.05	**1.17**	0.82	1.66	**1.13**	0.64	1.99

4th quartile																					
50-59 years	**0.79**	0.53	1.17	**0.69**	0.46	1.05	**0.99**	0.63	1.54	**0.90**	0.62	1.31	**0.90**	0.58	1.38	**0.78**	0.50	1.23	**1.10**	0.56	2.16
60-64 years	**0.69**	0.46	1.05	**0.61**	0.35	1.04	**1.51**	0.87	2.60	**0.65**	0.38	1.12	**0.96**	0.53	1.74	**0.97**	0.57	1.66	**0.89**	0.39	2.03
Non smoker	**0.79**	0.40	1.56	**0.64**	0.31	1.32	**1.53**	0.76	3.04	**1.22**	0.66	2.24	**0.67**	0.30	1.52	**0.74**	0.39	1.41	**1.29**	0.47	3.53
Smoker	**0.70**	0.50	0.99	**0.66**	0.46	0.95	**1.05**	0.71	1.57	**0.77**	0.53	1.10	**0.97**	0.66	1.43	**0.91**	0.61	1.37	**0.93**	0.51	1.71
BMI <25.0	**0.83**	0.50	1.36	**0.74**	0.45	1.21	**1.10**	0.65	1.86	**1.07**	0.66	1.74	**1.05**	0.61	1.80	**0.69**	0.42	1.13	**0.61**	0.32	1.18
BMI >25.0	**0.68**	0.46	1.00	**0.60**	0.39	0.93	**1.24**	0.79	1.94	**0.73**	0.49	1.10	**0.83**	0.52	1.30	**1.01**	0.63	1.62	**2.12**	0.82	5.47
Sedentary	**0.81**	0.56	1.18	**0.75**	0.50	1.14	**1.10**	0.71	1.70	**1.04**	0.71	1.53	**0.84**	0.54	1.30	**0.81**	0.52	1.26	**1.08**	0.56	2.07
Active at work	**0.62**	0.36	1.07	**0.55**	0.32	0.94	**1.33**	0.77	2.29	**0.65**	0.38	1.10	**1.10**	0.62	1.95	**0.96**	0.55	1.67	**0.88**	0.37	2.10
Not working	**0.84**	0.47	1.50	**0.86**	0.44	1.67	**1.59**	0.75	3.33	**0.68**	0.37	1.23	**0.60**	0.28	1.33	**0.92**	0.48	1.78	**0.98**	0.36	2.69
Working	**0.65**	0.45	0.95	**0.58**	0.39	0.86	**1.08**	0.73	1.62	**0.90**	0.63	1.29	**1.01**	0.69	1.50	**0.80**	0.53	1.19	**1.00**	0.55	1.85

Total number of persons in each stratum: *50-59 years* = 40 085, n cases = 374 | *60-64 years* = 14 560, n cases = 155 | *Non smoker* = 19 269, n cases = 134 | *Smoker* = 35 376, n cases = 395 | *BMI <25* = 23 648, n cases = 232 | *BMI>25* = 30 997, n cases = 297 | *Sedentary work* = 31 697, n cases = 345 | *Active at work* = 22 948, n cases = 184 | *Not working* = 12 041, n cases = 146 | *Working* = 42 604, n cases = 383
